# Suppression of salt-enhanced apoplastic flow by salicylic acid in rice

**DOI:** 10.1007/s12298-026-01733-3

**Published:** 2026-03-18

**Authors:** Md. Asadulla Al Galib, Maoxiang Zhao, Toshiyuki Nakamura, Yoshimasa Nakamura, Yoshihiko Hirai, Yoshitaka Nakashima, Shintaro Munemasa, Izumi C. Mori, Yoshiyuki Murata

**Affiliations:** 1 Graduate School of Environmental and Life Science, Okayama University, Okayama, 700-8530 Japan; 2 Institute of Plant Science and Resources, Okayama University, Kurashiki, 710-0046 Japan

**Keywords:** Apoplastic flow, Salicylic acid, Rice, Salinity, Trisodium-8-hydroxy-1,3,6-pyrenetrisulphonic acid

## Abstract

Salinity enhances apoplastic flow, resulting in an increment of Na^+^ uptake and a lower K^+^/Na^+^ ratio. Salicylic acid (SA) plays an important role in improving salinity tolerance in plants. The effect of exogenous SA on apoplastic flow in salt-treated rice seedlings was studied using an apoplastic tracer, 8-hydroxy-1,3,6-pyrenetrisulphonic acid (PTS) in light. Application of NaCl at 25 mM to the hydroponic solution significantly increased PTS uptake, while 25 mM NaCl did not affect seedling growth. Application of 25 mM NaNO_3_ increased PTS uptake to the same degree. Salinity significantly increased sodium (Na^+^) content but had no significant effect on potassium (K^+^) content, resulting in a lower K^+^/Na^+^ ratio. The application of SA at 0.05 mM and 0.1 mM to the hydroponic solution reduced Na-enhanced PTS uptake. Salicylic acid at 0.05 mM and 0.1 mM significantly reduced Na^+^ content and slightly increased K^+^ content in the shoots of rice seedlings, resulting in a higher K^+^/Na^+^ ratio. However, SA at up to 0.1 mM did not increase SA contents in shoots under salt stress. These results suggest that exogenous SA reduces Na^+^ uptake by suppressing Na^+^-enhanced apoplastic flow in rice seedlings. These findings provide insight into modulation of Na^+^ transport pathways from roots to shoots by SA and may allow us to utilize brackish water for rice cultivation and to improve salt-tolerant rice through suppression of salt-enhanced apoplastic flow by chemicals such as salicylic acid.

**Supplementary Information:**

The online version contains supplementary material available at 10.1007/s12298-026-01733-3.

## Introduction

Salinity is a major abiotic stress that severely limits agricultural productivity by inducing osmotic stress and ion toxicity (Hasegawa et al. 1986; Wang et al. 2022). High salt concentrations in the plant root zone cause osmotic stress, which reduces water uptake, disrupts cell membranes, and impairs plant growth and metabolism (Munns 2002; Wang et al. 2022). Sodium (Na^+^) and chloride (Cl^−^) ions are the most common ions in saline soils (Yoshida 1981). The excessive accumulation of Na^+^ in the plant cells disrupts ion homeostasis. As a result, K⁺/Na⁺ ratio in the plant cells decreases, which negatively affects enzyme activity and essential cellular functions (Niu et al. 1995).

Rice (*Oryza sativa* L.) is highly sensitive to salinity, especially at the early growth stage (Yoshida 1981). Nipponbare (*Oryza sativa* L.) is a salt-sensitive *japonica* rice variety commonly used as a model rice cultivar for studying abiotic and biotic stress responses (Wang et al. 2012). Rice plants use an apoplastic bypass pathway for Na^+^ transport which allows toxic levels of Na^+^ accumulation in the leaves (Yeo et al. 1987; Garcia et al. 1997), whereas most plants transport Na^+^ to the root xylem through the symplastic pathway, including transporters (Munns et al. 2002). Apoplastic flow in rice was higher than that in other cereals (Faiyue et al. 2012). Moreover, apoplastic flow was highly correlated to Na^+^ uptake in rice but not in wheat (Garcia et al. 1997) and significant changes in Na^+^ contents in shoots of wheat and barley exposed to 50 mM NaCl were not detected (Lu and Fricke 2023). Hence, apoplastic flow more significantly contributes to Na⁺ transport to shoots in rice than in wheat and barley. In addition, a non-toxic, water-soluble fluorescent dye 8-hydroxypyrene-1,3,6-trisulfonic acid (PTS) is widely used to trace apoplastic flow in rice seedlings (Yeo et al. 1987; Garcia et al. 1997; Sobahan et al. 2009, 2012).

Apoplastic flow plays a major role in Na^+^ uptake in rice (Yeo et al. 1987; Sobahan et al. 2009). A large portion of Na⁺ enters the shoots through the apoplastic pathway in rice hydroponically grown in 25–50 mM saline conditions (Faiyue et al. 2010). Various exogenous chemicals such as polyethylene glycol (PEG), silicon (Si), proline, betaine, and chitosan have been reported to reduce the apoplastic flow in rice (Yeo et al. 1999; Ochiai and Matoh 2004; Sobahan et al. 2009; Zhao et al. 2025).

Among potential growth regulators, salicylic acid (SA) is a naturally occurring phenolic compound abundant in rice plants (Silverman et al. 1995; Yang et al. 2004). Salicylic acid mitigates the detrimental effects of salinity in rice by improving plant water status, ion homeostasis, and antioxidant enzyme activities (Jayakannan et al. 2015; Pai and Sharma 2023). However, it is unclear whether exogenous SA modulates apoplastic flow in rice seedlings.

In this study, we investigated the effects of exogenous SA on salt-enhanced apoplastic flow in rice seedlings.

## Materials and methods

### Plant materials

Rice seedlings were grown as described by Sobahan et al. (2009). Rice seeds were germinated in water-filled Petri dishes under 12-h light (30 °C) and 12-h dark (25 °C) with white fluorescent light (80 µmol m^− 2^ s^− 1^) in a growth chamber. After 7 days, seedlings were transferred to pots containing Kimura B nutrient solution (0.36 mM (NH_4_)_2_SO_4_, 0.55 mM MgSO_4_·7H_2_O, 0.18 mM KNO_3_, 0.18 mM KH_2_PO_4_, 0.36 mM Ca(NO_3_)_2_·4H_2_O, 0.05 mM Fe(III)EDTA, 0.05 mM H_3_BO_3_, 0.01 mM MnCl_2_·4H_2_O, 0.32 µM CuSO_4_·5H_2_O, 0.52 µM Na_2_MoO_4_·2H_2_O and 0.77 µM ZnSO_4_·7H_2_O) and grown under the same conditions. The hydroponic solution was changed every two days. The relative humidity was maintained at 65 ± 2% (light) and 55 ± 2% (dark) in the growth chamber. Seedlings were cultured for 14 d and were used as samples in further experiments. Five 3-week-old rice seedlings were placed in a 1-L plastic pot supplemented with fresh hydroponic solution. Each experiment was independently repeated three times (*n* = 3), that is, each data point was obtained from 15 plants.

## Growth

Shoot length (cm), root length (cm), shoot fresh weight (g plant^− 1^), and root fresh weight (g plant^− 1^) were measured after the seedlings were treated with and without 25 mM NaCl in the presence or absence of 0.05 mM and 0.1 mM SA for 7 days.

## Measurement of apoplastic flow

The apoplastic flow was measured using an apoplastic tracer, 8-hydroxy-1,3,6-pyrenetrisulphonic acid (PTS), as described by Sobahan et al. (2009). In short, rice seedlings were placed in a hydroponic solution containing 300 mg L^− 1^ of PTS, with and without 25 mM NaCl in the presence or the absence of 0.05 mM and 0.1 mM SA for 12-h under light (80 µmol m^− 2^ s^− 1^). Then, whole shoots were collected and kept at -20 °C for 24-h. The entire shoots were used to extract tissue sap using the freeze-thaw method. The PTS concentration in the sap was measured from fluorescence intensity at 403 nm excitation and 510 nm emission wavelength using a fluorescent spectrofluorometer (FP-8200, JASCO, Tokyo, Japan). Transpiration was measured gravimetrically and adjusted for water loss due to evaporation (Yeo et al. 1999). The apoplastic flow was calculated as a percentage of the total water flow using the method from Yeo et al. (1987). The fluorescence was not detectable in the sample extracted from the seedlings untreated with NaCl and PTS.

## Determination of Na^+^ and K^+^ content

Shoots from the treated seedlings were collected and weighed. The shoots were digested in 5 ml of 100 mM acetic acid at 90 °C for 2 h (Yeo et al. 1999). The Na^+^ and K^+^ contents in the shoot extracts were measured using atomic absorption spectroscopy (model 6100, Hitachi, Japan).

## Determination of salicylic content

The salicylic acid content was measured in the shoots of rice seedlings using the method of Meuwly and Métraux (1993) with modifications. Around 1.0 g of fresh shoot tissue was used to determine salicylic acid content. In this study, 250 ng of *O*-anisic acid was used as an internal standard and 25 µg of para-hydroxybenzoic acid was used as an extraction carrier.

The salicylic acid (SA) content was determined using high-performance liquid chromatography (HPLC). The C18 column (4.6 mm i.d. х 250 mm х 5 μm, GL Sciences, Tokyo, Japan) was operated at 25 °C. The mobile phase consisted of 70% methanol and 0.1% acetic acid at a ratio of 60:40 (v/v). The injection volume was 10 µL. Salicylic acid was quantified fluorimetrically (excitation wavelength: 305 nm; emission wavelength: 407 nm; RF-10AXL SHIMADZU fluorescence detector). A standard curve was obtained using 10, 20, 50, 100, and 200 ng/mL of SA.

### Statistical analysis

Data were statistically analyzed by Statistical Tools for Agriculture Research (STAR) 2.0.1 (2014), International Rice Research Institute (Manila, Philippines). The mean values were compared using the Tukey test with a 5% (*p* < 0.05) significance level.

## Results and discussion

### Effects of salicylic acid on shoot and root growth of rice seedlings treated with NaCl

**Fig. 1:**
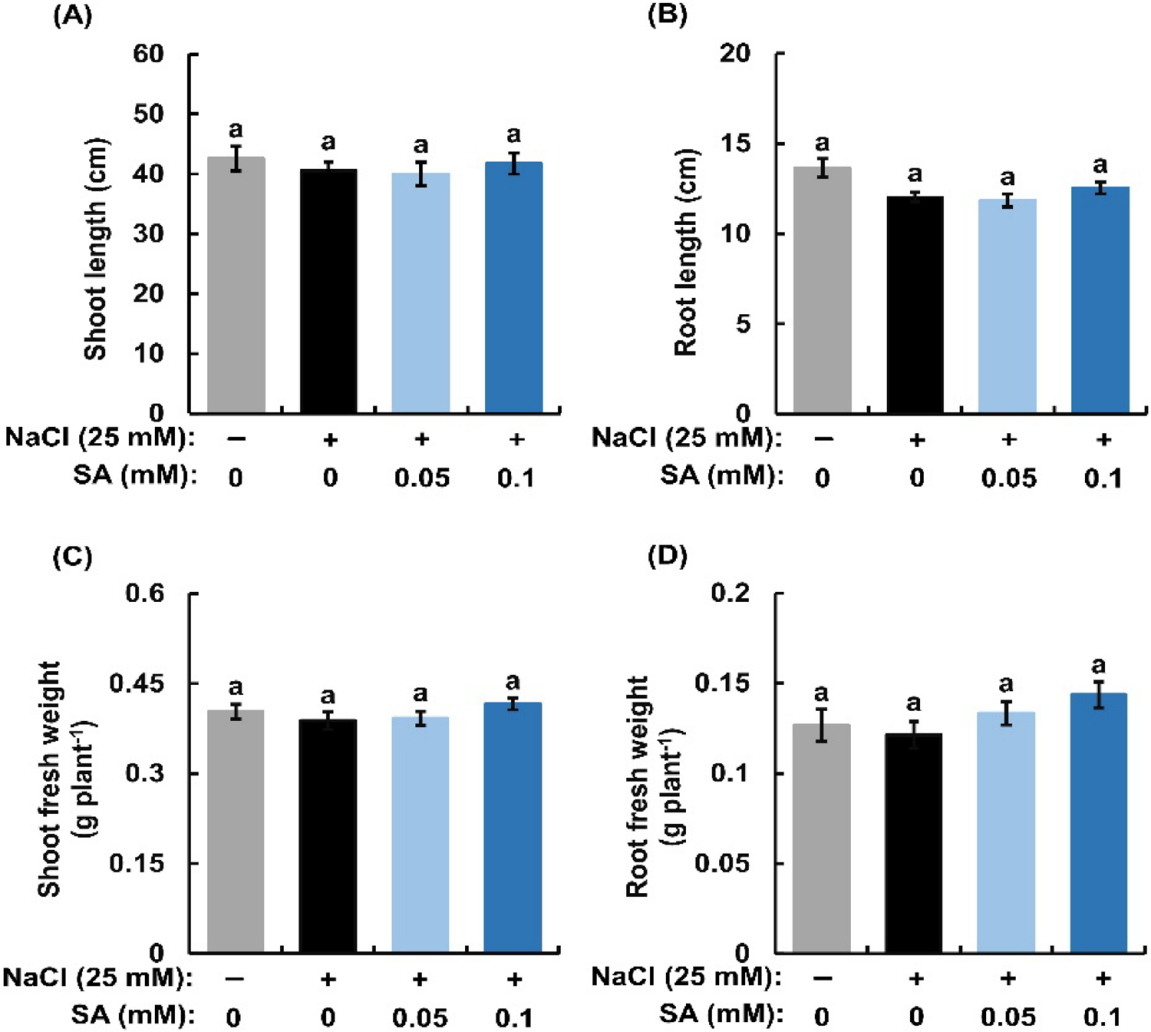
Effects of exogenous salicylic acid (SA) on shoot length (**A**), root length (**B**), shoot fresh weight (**C**) and root fresh weight (**D**) in salt-treated rice seedlings. Error bars represent the standard error of the mean (*n* = 3). Bars with different letters are significantly different at *p* < 0.05

We measured the growth of rice seedlings treated with or without NaCl (25 mM) in the presence or absence of 0.05 mM and 0.1 mM SA (Fig. 1). Salinity (25 mM NaCl) did not inhibit the growth of rice seedlings in the absence of SA. Moreover, SA at 0.05 mM and 0.1 mM did not affect the growth of rice seedlings treated with 25 mM NaCl. Hence, we employed this experimental condition so that differences in results are not foremost attributed to the difference in growth or mass of rice seedlings.

### Apoplastic flow in rice seedlings treated with NaCl

**Fig. 2:**
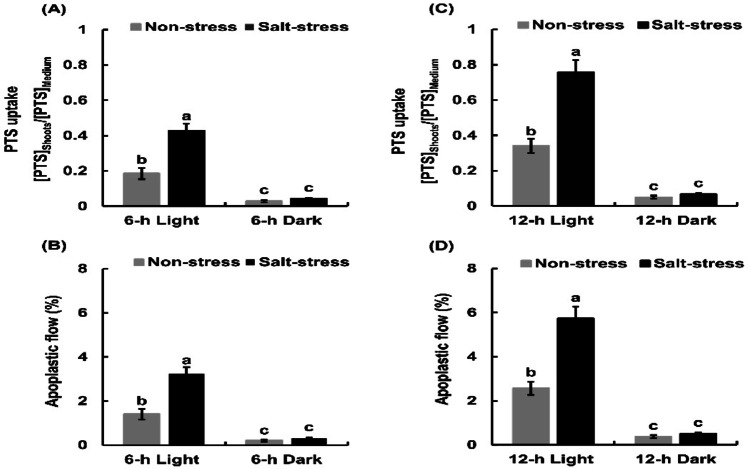
Effects of light and incubation time on PTS uptake and apoplastic flow in the shoots of rice seedlings. [PTS]_Shoots_ represents the PTS concentration in the cell sap of the shoot and [PTS]_Medium_ denotes the PTS concentration in the external medium. Apoplastic flow was calculated based on PTS uptake data. Error bars represent the standard error of the mean (*n* = 3). Bars with different letters are significantly different at *p* < 0.05

We measured PTS uptake in the rice seedlings treated with NaCl in the light and in the dark for 6 and 12-h. The PTS uptake and apoplastic flow were significantly higher under light conditions than under dark conditions (Fig. 2). The seedlings took up more PTS when 25 mM NaCl was added to the hydroponic solution than when no NaCl (Fig. 2A, C). The PTS uptake and apoplastic flow in the NaCl-treated rice seedlings were higher after 12-h incubation (Fig. 2C, D) than after 6-h incubation (Fig. 2A, B). Therefore, seedlings were incubated for 12-h under light conditions in the subsequent experiments.

**Fig. 3:**
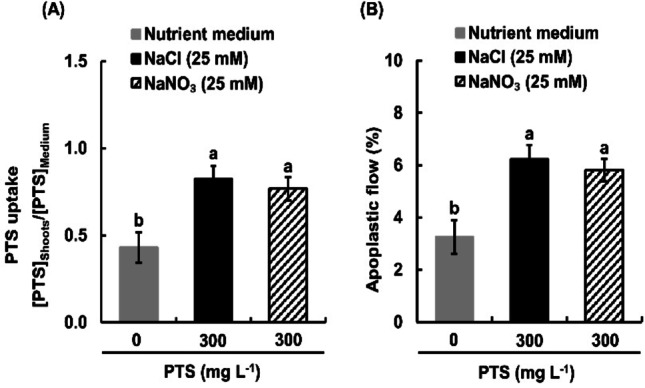
Effects of sodium (Na^+^) on PTS uptake (**A**) and apoplastic flow (**B**) in salt-treated rice seedlings. [PTS]_Shoots_ represents the PTS concentration in the cell sap of the shoot and [PTS]_Medium_ denotes the PTS concentration in the external medium. Apoplastic flow was calculated based on PTS uptake data. Error bars represent the standard error of the mean (*n* = 3). Bars with different letters are significantly different at *p* < 0.05

To investigate whicher the apoplastic flow was increased by Na^+^ or Cl^−^, we measured PTS uptake in shoots of NaCl- and NaNO_3_-treated rice seedlings (Fig. 3A, B). Both 25 mM NaCl and 25 mM NaNO_3_ significantly increased PTS uptake by 91.4% and by 80.1% (Fig. 3A) and apoplastic flow by 91.6% and by 80.5% (Fig. 3B) in rice seedlings. However, there was no significant difference in PTS uptake or in apoplastic flow between NaCl- and NaNO_3_-treated seedlings. These results suggest that the increment of PTS uptake and apoplastic flow is attributed to Na^+^ rather than anions. We used NaCl at 25 mM in order to examine the effects of SA on salinity-enhanced apoplastic flow in subsequent experiments.

### Suppression of salinity-enhanced apoplastic flow by salicylic acid in rice seedlings

**Fig. 4:**
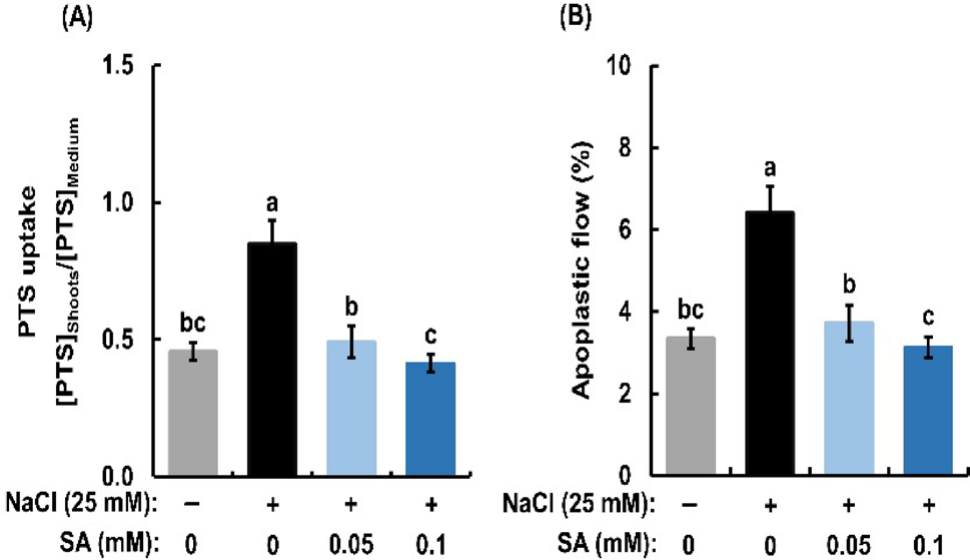
Effects of exogenous salicylic acid (SA) on PTS uptake (**A**) and apoplastic flow (**B**) in salt-treated rice seedlings. [PTS]_Shoots_ represents the PTS concentration in the cell sap of the shoot and [PTS]_Medium_ denotes the PTS concentration in the external medium. Apoplastic flow was calculated based on PTS uptake data. Error bars represent the standard error of the mean (*n* = 3). Bars with different letters are significantly different at *p* < 0.05

Application of 25 mM NaCl significantly increased PTS uptake and apoplastic flow to the same extent in rice seedlings (Fig. 4A, B), indicating that enhanced apoplastic flow under salinity promotes greater PTS uptake. Exogenous SA at 0.05 mM and at 0.1 mM reduced the increased PTS uptake by 42.1% and 51.4% (Fig. 4A) and the increased apoplastic flow by 42.1% and 51.2% (Fig. 4B) in the salt-treated rice seedlings.

**Fig. 5:**
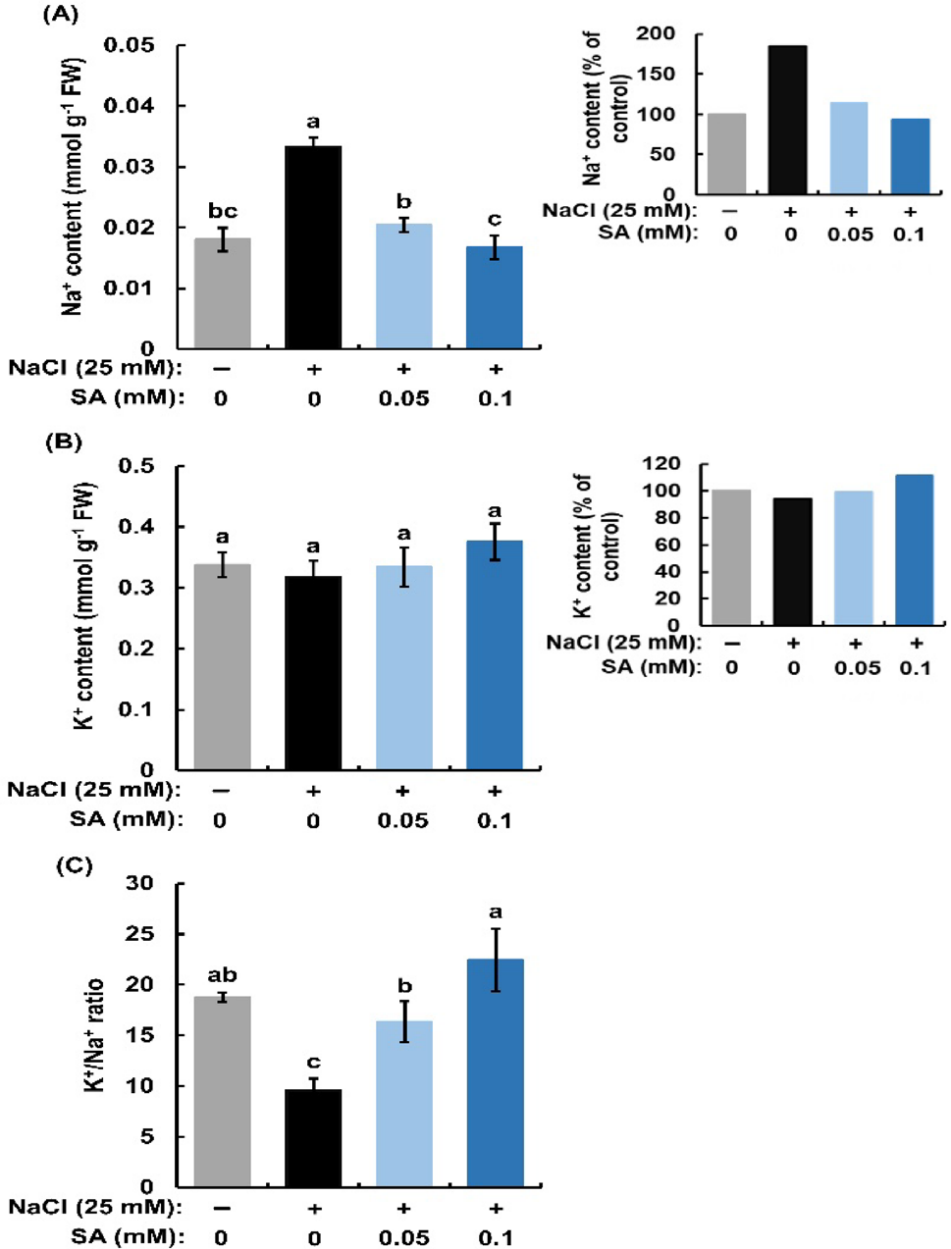
Effects of exogenous salicylic acid (SA) on sodium (Na^+^) content (**A**), potassium (K^+^) content (**B**) and K^+^/Na^+^ ratio (**C**) based on (**A**) and (**B**) in salt-treated rice seedlings. Error bars represent the standard error of the mean (*n* = 3). Bars with different letters are significantly different at *p* < 0.05

Contents of Na^+^ and K^+^ were measured in the shoots of rice seedlings treated with and without 25 mM NaCl for 12-h (Fig. 5A, B). Salinity significantly increased Na⁺ content (Fig. 5A) while salinity did not affect K⁺ content in rice seedlings (Fig. 5B), suggesting that Na^+^ uptake is enhanced by increment of apoplastic flow. The exogenous SA at 0.05 mM and at 0.1 mM reduced Na⁺ content (Fig. 5A) but did not change K⁺ content in the shoots of salt-treated rice seedlings (Fig. 5B).

The K^+^/Na^+^ ratio (Fig. 5C) was calculated from K^+^ and Na^+^ contents shown in Fig. 5A, B. Salinity significantly lowered the K^+^/Na^+^ ratio in the shoots of rice seedlings from 18.7 to 9.5. Salicylic acid at 0.05 mM and at 0.1 mM significantly improved K^+^/Na^+^ ratio in the shoots of salinity-treated seedlings (Fig. 5C), which is agreement with previous studies (Jayakannan et al. 2015; Pai and Sharma 2023) showed that SA improves salt tolerance by enhancing K⁺/Na⁺ ratio and reducing Na⁺ uptake. Moreover, our findings suggest that exogenous SA reduces Na^+^ uptake by suppressing the salinity-enhanced apoplastic flow, resulting in a higher K⁺/Na⁺ ratio.

### Salicylic acid content in rice seedlings

**Fig. 6:**
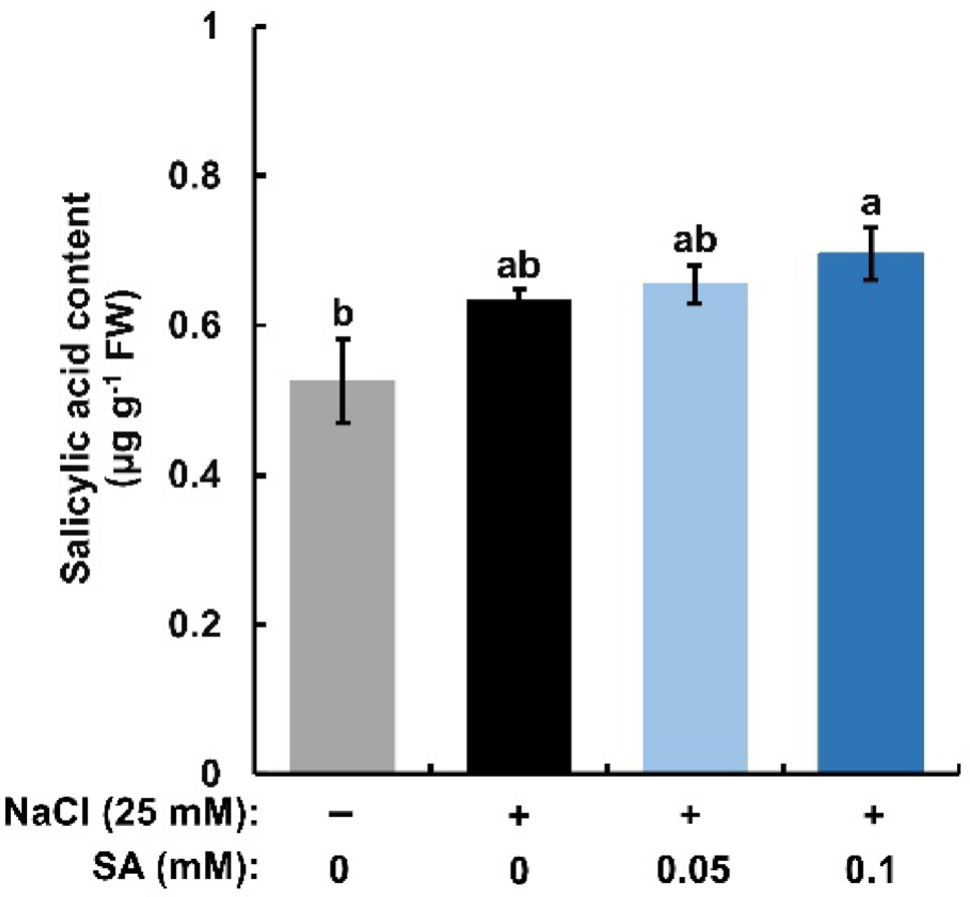
Effects of exogenous salicylic acid (SA) on salicylic acid contents in salt-treated rice seedlings. Error bars represent the standard error of the mean (*n* = 3). Bars with different letters are significantly different at *p* < 0.05

Salicylic acid in the shoots of the rice seedlings was quantified (Fig. 6). The treatment with 25 mM NaCl did not significantly change the SA content in the shoots of rice seedlings in the absence of SA in the medium. In the presence of 25 mM NaCl in the medium, exogenous SA significantly increased SA contents at 0.1 mM but not at 0.05 mM. Salicylic acid can move both symplastically and apoplastically in plants (Kachroo et al. 2020; Kim and Lim 2023). However, SA at 0.05 mM and 0.1 mM suppressed the salinity-enhanced apoplastic flow (Fig. 4) and the apoplastic flow in the presence of 0.05 mM SA was higher than that in the presence of 0.1 mM SA (Fig. 4). The increment of SA contents is not likely to be due to apoplastic transport of SA.

Treatment with SA at 0.1 mM for 96 h increased pectin contents and lignin contents in the roots of rice seedlings (Pan et al. 2021) while SA at 0.1 mM significantly suppressed the Na^+^-enhanced apoplastic flow within 12 h and NaCl at 25 mM doubled apoplastic flow within 6 h in rice seedlings (Fig. 2; Sobahan et al. 2009). Hence, apoplastic flow is likely to be more quickly changed by Na^+^ and SA than the composition of pectin and lignin is. These results suggest that the suppression of Na^+^-enhanced apoplastic flow by SA is not due to reinforcement of cell wall barriers. Moreover, treatments with SA at up to 0.1 mM did not significantly increase SA contents in the shoots of salt-stressed seedlings (Fig. 6), suggesting that suppression of stomatal transpiration is not dominantly involved in the suppression of apoplastic flow by SA.

We tested the effects of probenazole, which can increase SA contents in rice plants (Sato et al. 2013), although thebiosynthesis pathway of SA in rice is not fully understood (Duan et al. 2014; Wang et al. 2024). Probenazole (PBZ) at up to 0.1 mM did not affect growth in rice seedlings (Fig. S1). Both 0.1 mM PBZ and 0.1 mM SA suppressed the Na^+^-enhanced apoplastic flow (Fig. S2), although neither PBZ nor SA significantly increased SA contents in the shoots of salt-stressed rice (Fig. S3). These results suggest that SA absorbed into the shoots does not suppress apoplastic flow.

## Conclusion

We can conclude that SA suppresses salt-enhanced apoplastic flow, thereby reducing Na^+^ influx and improving ionic balance in rice seedlings. Furthermore, these findings provide insight into chemical modulation of Na^+^ transport pathways from roots to shoots under weak salt stress condition and may allow us to utilize brackish water for salt-sensitive rice such as Nipponbare.

## Supplementary Information

Below is the link to the electronic supplementary material.

Supplementary Material 1

Supplementary Material 2

Supplementary Material 3

Supplementary Material 4

## Data Availability

The authors declare that all data generated or analyzed during this investigation are available from the corresponding author on request.

## References

[CR1] Duan L, Liu H, Li X et al (2014) Multiple phytohormones and phytoalexins are involved in disease resistance to *Magnaporthe oryzae* invaded from roots in rice. Physiol Plant 152:486–500. 10.1111/ppl.12192 24684436 10.1111/ppl.12192

[CR2] Faiyue B, Al-Azzawi MJ, Flowers TJ (2010) The role of lateral roots in bypass flow in rice (*Oryza sativa* L). Plant Cell Environ 33:702–716. 10.1111/j.1365-3040.2009.02078.x 19930130 10.1111/j.1365-3040.2009.02078.x

[CR3] Faiyue B, Al-Azzawi MJ, Flowers TJ (2012) A new screening technique for salinity resistance in rice (*Oryza sativ*a L.) seedlings using bypass flow: bypass flow and screening for salt tolerance. Plant Cell Environ 35:1099–1108. 10.1111/j.1365-3040.2011.02475.x 22171658 10.1111/j.1365-3040.2011.02475.x

[CR4] Garcia A, Rizzo CA, Ud-din J et al (1997) Sodium and potassium transport to the xylem are inherited independently in rice, and the mechanism of sodium: potassium selectivity differs between rice and wheat. Plant Cell Environ 20:1167–1174. 10.1046/j.1365-3040.1997.d01-146.x

[CR5] Hasegawa PM, Bressan RA, Handa AK (1986) Cellular mechanisms of salinity tolerance. HortScience 21:1317–1324. 10.21273/HORTSCI.21.6.1317

[CR6] Jayakannan M, Bose J, Babourina O (2015) Salicylic acid in plant salinity stress signalling and tolerance. Plant Growth Regul 76:25–40. 10.1007/s10725-015-0028-z

[CR7] Kachroo P, Liu H, Kachroo A (2020) Salicylic acid: transport and long-distance immune signaling. Curr Opin Virol 42:53–57. 10.1016/j.coviro.2020.05.008 32544865 10.1016/j.coviro.2020.05.008

[CR8] Kim TJ, Lim GH (2023) Salicylic acid and mobile regulators of systemic immunity in plants: transport and metabolism. Plants 12:1013. 10.3390/plants12051013 36903874 10.3390/plants12051013 PMC10005269

[CR9] Lu Y, Fricke W (2023) Diurnal changes in apoplast bypass flow of water and ions in salt-stressed wheat (*Triticum aestivum* L.) and barley (*Hordeum vulgare* L). Physiol Plant 175:e13955. 10.1111/ppl.13955 37323067 10.1111/ppl.13955

[CR10] Meuwly P, Me´traux JP (1993) Ortho-anisic acid as internal standard for the simultaneous quantitation of salicylic acid and its putative biosynthetic precursors in cucumber leaves. Anal Biochem 214:500–505. 10.1006/abio.1993.1529 8109740 10.1006/abio.1993.1529

[CR11] Munns R (2002) Comparative physiology of salt and water stress. Plant Cell Environ 25:239–250. 10.1046/j.0016-8025.2001.00808.x 11841667 10.1046/j.0016-8025.2001.00808.x

[CR12] Munns R, Husain S, Rivelli AR et al (2002) Avenues for increasing salt tolerance of crops, and the role of physiologically based selection traits. Plant Soil 247:93–105. 10.1023/A:1021119414799

[CR13] Niu X, Bressan RA, Hasegawa PM et al (1995) Ion homeostasis in NaCl stress environments. Plant Physiol 109:735–742. 10.1104/pp.109.3.735 12228628 10.1104/pp.109.3.735 PMC161372

[CR14] Ochiai K, Matoh T (2004) Alleviation of salinity damage to rice plants by the use of polyethylene glycols (PEGs) through reduction of Na^+^ transport to shoots. Soil Sci Plant Nutr 50:129–133. 10.1080/00380768.2004.10408460

[CR15] Pai R, Sharma PK (2023) Exogenous application of salicylic acid mitigates salt stress in rice seedlings by regulating plant water status and preventing oxidative damage. Environ Exp Biol 20:193–204. 10.22364/eeb.20.18

[CR16] Pan J, Guan M, Xu P et al (2021) Salicylic acid reduces cadmium (Cd) accumulation in rice (*Oryza sativa* L.) by regulating root cell wall composition via nitric oxide signaling. Sci Total Environ 25:797. 10.1016/j.scitotenv.2021.149202 10.1016/j.scitotenv.2021.149202 34346363

[CR17] Sato Y, Kato H, Endo A et al (2013) Probenazole promotes root growth and suppresses expression of pathogenesis-related proteins in roots of rice seedlings. Res Bull NARO Hokkaido Agric Res Cent 198:69–81

[CR18] Silverman P, Seskar M, Kanter D et al (1995) Salicylic acid in rice (biosynthesis, conjugation, and possible role). Plant Physiol 108:633–639. 10.1104/pp.108.2.633 12228500 10.1104/pp.108.2.633 PMC157383

[CR20] Sobahan MA, Arias CR, Okuma E et al (2009) Exogenous proline and glycinebetaine suppress apoplastic flow to reduce Na^+^ uptake in rice seedlings. Biosci Biotechnol Biochem 73:2037–2042. 10.1271/bbb.90244 19734659 10.1271/bbb.90244

[CR19] Sobahan MA, Akter N, Ohno M et al (2012) Effects of exogenous proline and glycinebetaine on the salt tolerance of rice cultivars. Biosci Biotechnol Biochem 76:1568–1570. 10.1271/bbb.120233 22878180 10.1271/bbb.120233

[CR22] Wang H, Ahan J, Wu ZH et al (2012) Alteration of nitrogen metabolism in rice variety ‘Nipponbare’ induced by alkali stress. Plant Soil 355:131–147. 10.1007/s11104-011-1086-2

[CR21] Wang CF, Han GL, Yang ZR et al (2022) Plant salinity sensors: current understanding and future directions. Front Plant Sci 13:859224. 10.3389/fpls.2022.859224 35463402 10.3389/fpls.2022.859224 PMC9022007

[CR23] Wang Z, Yang G, Zhang D et al (2024) Isochorismate synthase is required for phylloquinone, but not salicylic acid biosynthesis in rice. Abiotech 5(4):488–496. 10.1007/s42994-024-00166-4 39650133 10.1007/s42994-024-00166-4 PMC11624176

[CR24] Yang Y, Qi M, Mei C (2004) Endogenous salicylic acid protects rice plants from oxidative damage caused by aging as well as biotic and abiotic stress. Plant J 40:909–919. 10.1111/j.1365-313X.2004.02267.x 15584956 10.1111/j.1365-313X.2004.02267.x

[CR26] Yeo AR, Yeo ME, Flowers TJ (1987) The contribution of an apoplastic pathway to sodium uptake by rice roots in saline conditions. J Exp Bot 38:1141–1153. 10.1093/jxb/38.7.1141

[CR25] Yeo AR, Flowers SA, Rao G et al (1999) Silicon reduces sodium uptake in rice (*Oryza sativa* L.) in saline conditions and this is accounted for by a reduction in the transpirational bypass flow. Plant Cell Environ 22:559–565. 10.1046/j.1365-3040.1999.00418.x

[CR27] Yoshida S (1981) Fundamentals of rice crop science. IRRI, Los Banos, Philippines

[CR28] Zhao M, Galib MA, Nakamura T et al (2025) Suppression of Na+ uptake via apoplastic flow by chitosan in rice. J Soil Sci Plant Nutr. 10.1007/s42729-025-02857-3

