# Supplementary figures and images for: Suppression of salt-enhanced apoplastic flow by salicylic acid in rice
Source: Physiol Mol Biol Plants. 2026 Mar 18;32(4):913–9. doi: 10.1007/s12298-026-01733-3 (PMC13125453; doi:10.1007/s12298-026-01733-3)

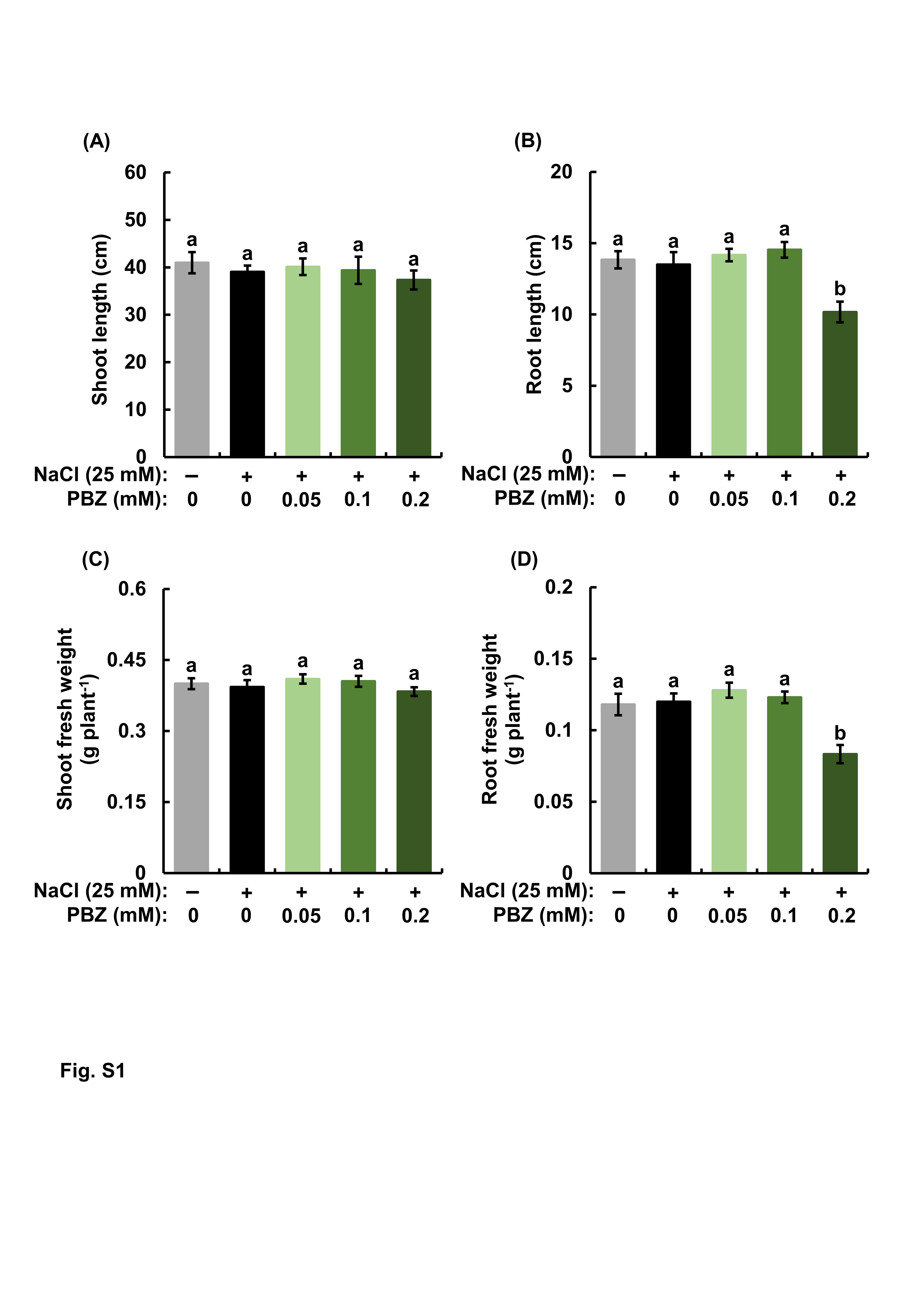

Supplement: Supplementary file 1 — Supplementary Material 1 [file 12298_2026_1733_MOESM1_ESM.tif]

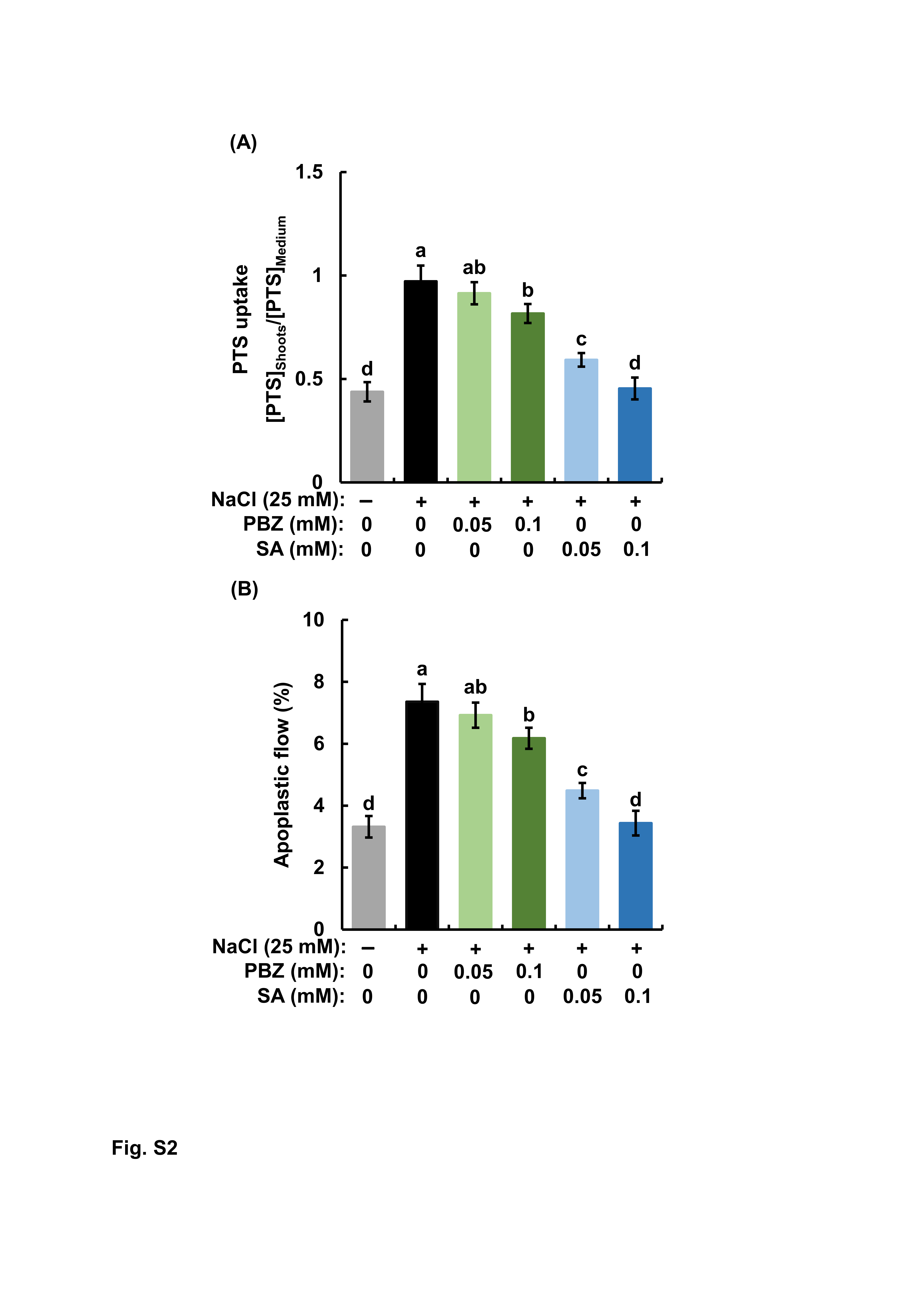

Supplement: Supplementary file 2 — Supplementary Material 2 [file 12298_2026_1733_MOESM2_ESM.tif]

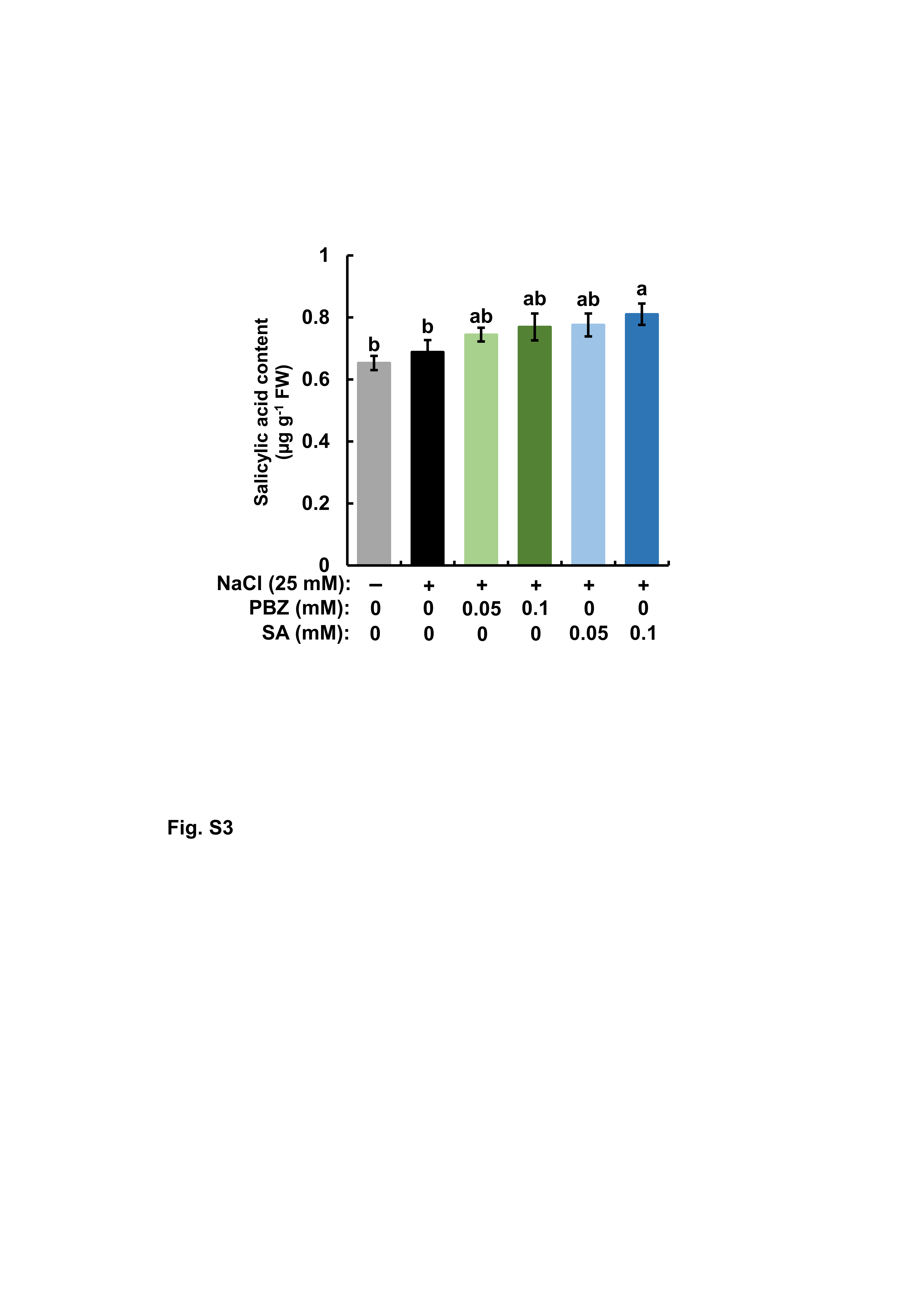

Supplement: Supplementary file 3 — Supplementary Material 3 [file 12298_2026_1733_MOESM3_ESM.tif]
