# Supplementary material for: Suppression of salt-enhanced apoplastic flow by salicylic acid in rice
Source: Physiol Mol Biol Plants. 2026 Mar 18;32(4):913–9. doi: 10.1007/s12298-026-01733-3 (PMC13125453; doi:10.1007/s12298-026-01733-3)
Supplement: Supplementary file 4 — Supplementary Material 4 [file 12298_2026_1733_MOESM4_ESM.docx]

**Supplementary Material**

Article title: Suppression of salt-enhanced apoplastic flow by salicylic acid in rice

Journal name: Physiology and Molecular Biology of Plants

Md. Asadulla Al Galib^1^ · Maoxiang Zhao^1^ · Toshiyuki Nakamura^1^ · Yoshimasa Nakamura^1^ · Yoshihiko Hirai^1^ · Yoshitaka Nakashima^1^ · Shintaro Munemasa^1^ · Izumi C. Mori^2^ · Yoshiyuki Murata^1,*^

^1^Graduate School of Environmental and Life Science, Okayama University, Okayama 700-8530, Japan

^2^Institute of Plant Science and Resources, Okayama University, Kurashiki, Okayama 710-0046, Japan

e-mail address of the corresponding author: muta@cc.okayama-u.ac.jp


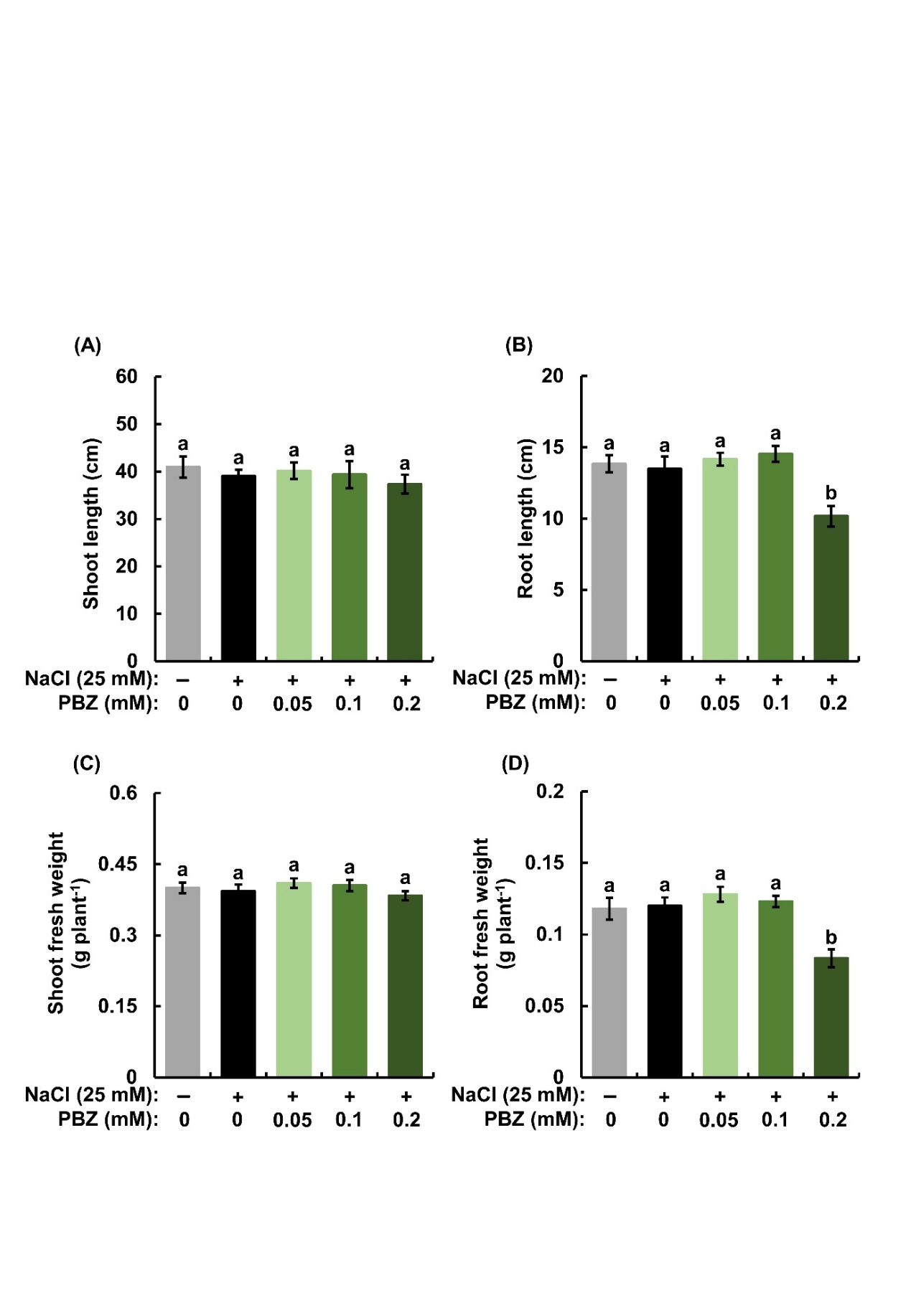


**Fig. S1** Effects of exogenous probenazole (PBZ) on shoot length (A), root length (B), shoot fresh weight (C), and root fresh weight (D) in salt-treated rice seedlings. Error bars represent the standard error of the mean (n=3). Bars with different letters are significantly different at *p* < 0.05


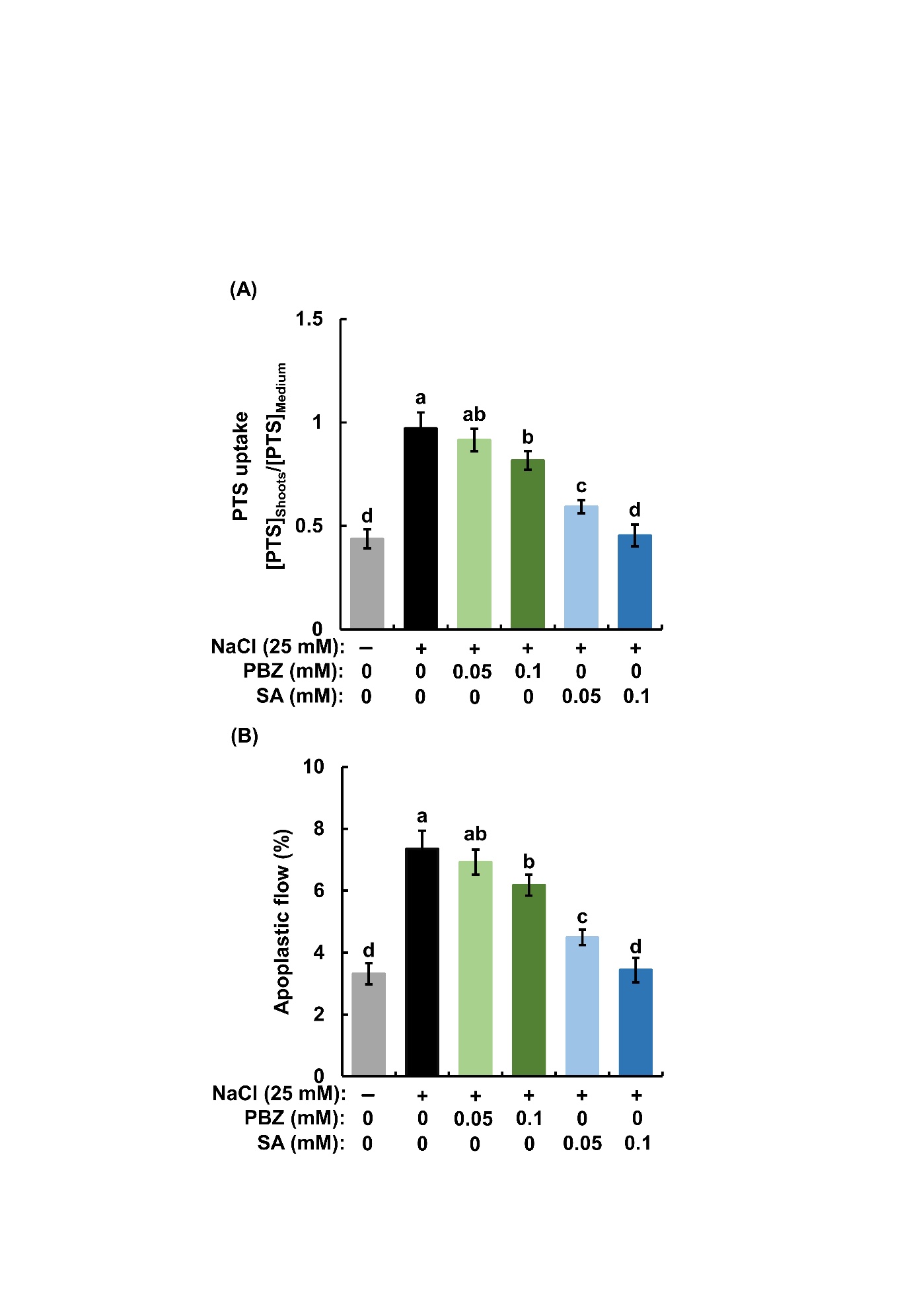


**Fig. S2** Effects of exogenous salicylic acid (SA) and probenazole (PBZ) on PTS uptake (A) and apoplastic flow (B) in salt-treated rice seedlings. [PTS]_Shoots_ represents the PTS concentration in the cell sap of the shoot and [PTS]_Medium_ denotes the PTS concentration in the external medium. Apoplastic flow was calculated based on PTS uptake data. Error bars represent the standard error of the mean (n=3). Bars with different letters are significantly different at *p* < 0.05


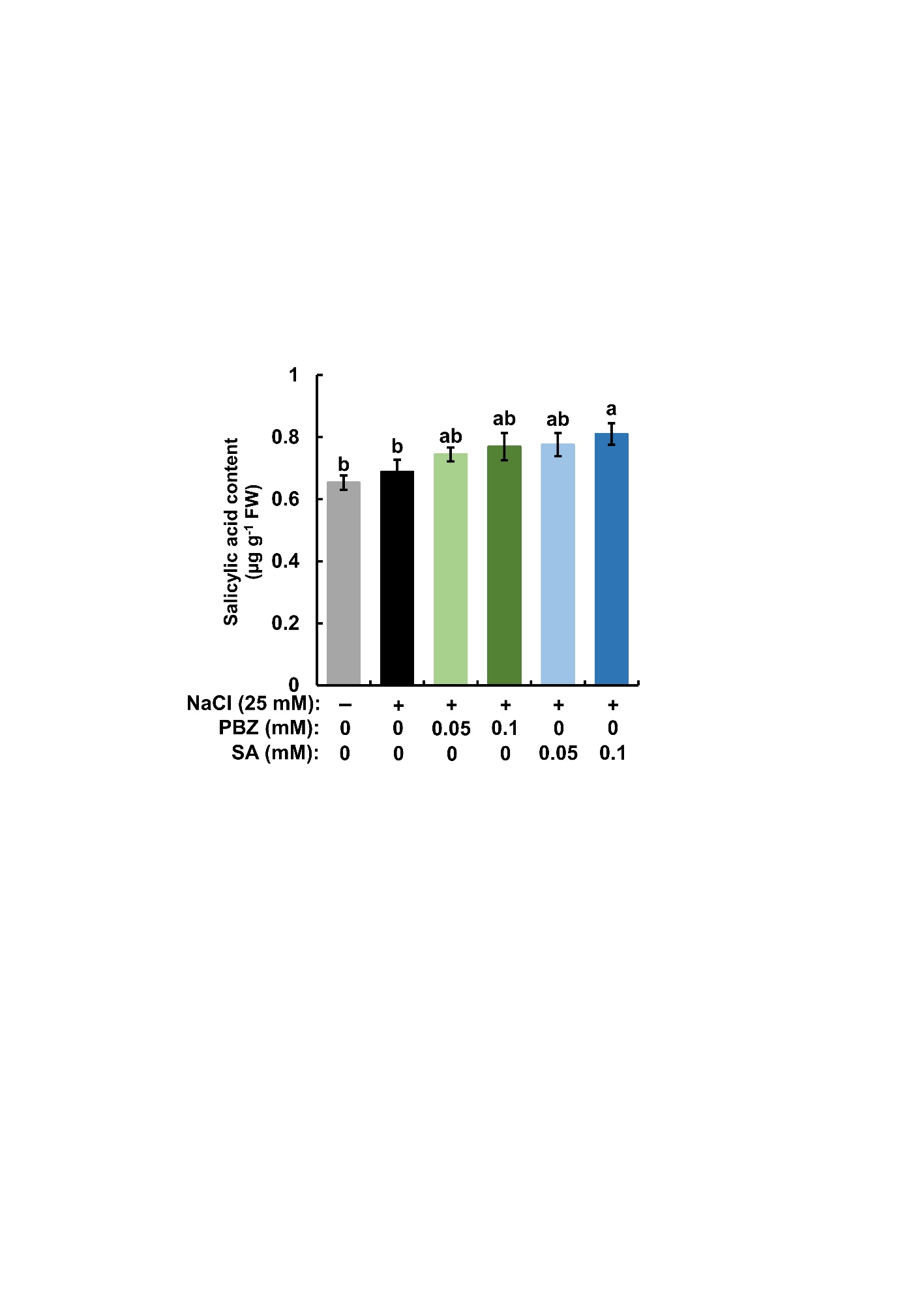


**Fig. S3** Effects of exogenous salicylic acid (SA) and probenazole (PBZ) on salicylic acid contents in salt-treated rice seedlings. Error bars represent the standard error of the mean (n=3). Bars with different letters are significantly different at *p* < 0.05
